# A stakeholder perspective on the necessary conditions for successfully implementing parenting interventions in Botswana

**DOI:** 10.3389/fpubh.2024.1355652

**Published:** 2024-09-13

**Authors:** Hlengiwe Gwebu, Tendai Elvis Mutembedza, Jacqueline Kilby, Jeldau Rieff, Styn Jamu, Lisa Jamu, Nomsa Monare, Mary Mosenke, Mmannyana Margaret Nonong, Babedi Ncaagae, Yulia Shenderovich, Jamie Lachman, Lucie Cluver, Catherine L. Ward

**Affiliations:** ^1^Department of Public Health, University of Fort Hare, East London, South Africa; ^2^Department of Psychology, University of Cape Town, Cape Town, South Africa; ^3^Centre for Social Science Research, University of Cape Town, Cape Town, South Africa; ^4^Department of Clinical, Educational and Health Psychology, University College London, London, United Kingdom; ^5^Stepping Stones International, Gaborone, Botswana; ^6^Ministry of Local Government and Rural Development, Department of Social Development, Family Welfare Services Division, Gaborone, Botswana; ^7^Wolfson Centre for Young People's Mental Health, Cardiff University, Cardiff, United Kingdom; ^8^Centre for the Development and Evaluation of Complex Interventions for Public Health Improvement (DECIPHer), School of Social Sciences, Cardiff University, Cardiff, United Kingdom; ^9^Centre for Evidence-Based Intervention, Department of Social Policy and Intervention, University of Oxford, Oxford, United Kingdom; ^10^Department of Psychiatry and Mental Health, University of Cape Town, J-Block, Groote Schuur Hospital Observatory, Cape Town, South Africa

**Keywords:** Botswana, parenting interventions, parent support programs, parent-child relationships, stakeholder perspectives, violence prevention

## Abstract

**Background:**

Encouraging positive parenting practices through evidence-based interventions is vital for the achievement of SDG target 16.2, which aims to eradicate all forms of violence against children while promoting their safety and mental wellbeing. As low- and middle- income countries increasingly adopt parenting programs, policymakers and implementers face the challenge of efficiently transporting, adapting, and implementing them across multiple settings.

**Purpose:**

This study seeks to evaluate the real-world experiences, challenges, and best practices in implementing parent support programs in Botswana.

**Method:**

A series of interviews with program implementers and stakeholders from governmental and non-governmental organizations were conducted. Key informants from governmental and non-governmental institutions were recruited through purposive and snowball sampling and 17 practitioners participated in the study. Data collection was carried out through online Zoom video conferencing at a convenient time and location for participants.

**Findings:**

The findings indicate several factors that contribute to the successful implementation of parenting programs in Botswana, including (a) enabling factors such as supportive policies, regulations and guidelines, (b) innovative factors such as capacity building, program adaptation and mixed method program delivery, (c) bridging factors through collaborations with skilled organizations, and (d) intra-organizational factors such as organizational resources, program sustainability, and support for program staff.

**Conclusion:**

No single organization or strategy can sustainably foster positive parenting support in Botswana. Instead, a collective and collaborative learning approach is necessary to develop lasting and scalable solutions.

## Introduction

Violence against children (VAC) remains a global issue, with the World Health Organization (WHO) acknowledging that it can take on many forms, including maltreatment, sexual violence, emotional abuse, and neglect (1). Children experience violence from strangers and acquaintances, including family and relatives. Often violence against children is meted in familiar settings, including homes, schools, and communities (1). Victims of VAC experience physical and psychological trauma that can, among others, result in physical disability and mental health disorders (2). The adverse health effects of VAC can be instant or delayed, short-lived or lifelong with varying degrees of intensity and sometimes resulting in death (3). VAC is connected to a combination of economic, cultural, and spatial factors, with prevalence rates highest in low and middle-income countries, particularly in Africa. Fortunately, parent support interventions have been shown to effectively curb VAC while improving children's wellbeing and development. Over the past decade, randomized trials in the Netherlands (4), New Zealand (5), and South Africa (6–8) have shown that evidence-based parenting programs can (1) prevent maltreatment of children and adolescents, (2) improve health across the lifespan, nutritional status, attendance at school and academic performance, social cohesion, psychosocial, and mental wellbeing, and (3) decrease the cycle of violence, including the perpetration of future violence, discriminatory norms, and harmful practices against children. However, the adoption and coverage of parenting interventions in low- and middle-income countries (LMICS) are slow relative to the burden of VAC. There is a growing uptake of parent support programs in LMICS with ongoing policy adjustments emphasizing child protection and wellbeing. Sustainable development goals (SDG) that seek to end violence, exploitation, abuse, and neglect of children, among many targets, have integrated their services into health and social welfare programs in developing countries (9, 10). Though with positive outcomes, the evaluation of parenting programs has focused chiefly on program-related outcomes. Given the persisting burden of VAC and the need to implement population-wide parent support programs, it is crucial to include policymakers and local stakeholders in parenting program evaluation to understand context specific operational aspects.

Insights from program implementers will be helpful to policymakers, funders, and other stakeholders in developing sustainable programs. We conducted this case study in Botswana to explore the experiences of parenting and social welfare program implementers to understand good practices, barriers, and facilitators of the sustainable implementation of parent support programs. This study was part of the Parenting for Lifelong Health Scale-Up of Parenting Evaluation Research, a multi-country study aiming at exploring the uptake, implementation, adaptation, evaluation and scaleup of Parenting for Lifelong Health programs in low- and middle-income countries (11). Botswana was one of the LMIC countries that participated in the SUPER study and was selected as a case study country among others. The Department of Social Protection (Family Welfare Services Division) in the Ministry of Local Government and Rural Development, in collaboration with Stepping Stones International, a non-government organization working in adolescent social protection joined the SUPER study. Detailed methods and procedures of the mother study are included in the study protocol published earlier (11).

## Aims and objectives

This study explored the experiences of participants involved in the implementation of parenting programs in Botswana, specifically focusing on the exploration, preparation, implementation, and sustainment phases. The research sought to identify barriers and facilitators for sustaining parent support programs in the country, as well as uncover valuable lessons learned during the implementation process and assess successful quality assurance measures. Furthermore, the study aimed to gather recommendations for sustaining and scaling up parent support programs in Botswana. The research questions that guided this study included exploring the experiences of participants in all phases of implementing parent support programs, identifying barriers and facilitators for sustaining such programs, uncovering lessons learned during the implementation process, and gathering recommendations for sustaining and scaling up parent support programs in Botswana. The purpose was to provide valuable insights and recommendations for improving the sustained implementation of parent support programs in Botswana.

## Conceptual framework

Our research questions were developed using the Exploration, Preparation, Implementation, Sustainment (EPIS) framework (12), a widely utilized tool in implementation studies and other fields that guides research implementation and outlines the stages of evidence-based interventions (13). The EPIS framework provides a comprehensive and systematic approach to understanding the key stages of implementing evidence-based interventions in real-world settings. It emphasizes the importance of exploring the needs and resources of the community, preparing for the implementation process, executing the intervention with fidelity, and sustaining the intervention's effectiveness over time. Table 1 shows a summary of research topics based on the EPIS model. These topics mainly covered participant involvement, experiences, lessons learnt, barriers, and facilitators across phases of the EPIS model.

**Table 1:** Summary of research topics based on the EPIS model.

**Exploration**	**Preparation**	**Implementation**	**Sustainment**
- Participant involvement in the exploration phase of implementing parent support programs. - Description of program adoption processes. - Lessons learnt: what works and what does not?	- Participant involvement in the preparation phase of implementing parent support programs. - Program preparation processes. - Lessons learnt: what works and what does not?	- Participant involvement in the implementation phase. - Description of the intervention and implementation process. - Assessment for quality and or effectiveness. - Lessons learnt: what works and what does not?	- Barriers and facilitators for sustaining parent support programs in Botswana. - Recommendations for sustaining and scaleup of parent support programs in Botswana.

Given the unique cultural, social, and economic contexts in Botswana, it is crucial to consider the perspectives of stakeholders involved in parenting interventions to ensure successful implementation. The EPIS framework allows for a thorough examination of the necessary conditions for implementing parenting interventions, acknowledging the diverse perspectives of stakeholders such as parents, caregivers, community leaders, and policymakers.

## Methods

We employed semi-structured interviews as our primary method of data collection. This approach provided a flexible yet systematic exploration of the subject matter, ensuring that we covered a wide range of perspectives and insights. Participants represented a diverse range of organizations, including governmental agencies, non-governmental organizations, and civil service entities involved in child and family health and welfare services in Botswana. All interviews were conducted in English by HG using a pilot tested, structured interview guide with prompts provided for probing. The structured interview guide ensured consistency in our data collection process, enabling us to conduct comparative analysis of responses across participants. Interviews took 45 min on average and field notes were made during such time. After interviews, participants were given a chance to verify information shared. The study focused participant opinions in their professional capacity about parenting program, it was a minimal risk study.

## Participants and procedures

The selection and engagement of 17 key informants for this study followed a rigorous and systematic process utilizing purposive and snowball sampling techniques in collaboration with local partners. Participants all adults above the age of 18 and mainly female, only 2/17 were male. Through targeted efforts, individuals with significant expertise and experience in family strengthening and parent support interventions were identified from various governmental, non-governmental, and civil service organizations in Botswana. This involved reaching out to organizations that offered social welfare and family support services, followed by contacting key personnel within these organizations to recommend suitable candidates to partake in the study as key informants. Subsequently, the selected individuals were approached through a series of telephonic and email communications to gauge their interest and willingness to participate in the research. This preliminary contact was further solidified during an initial video conferencing meeting, where the study's purpose, procedures, and expectations were elucidated. By adopting this targeted and methodical approach, the research team ensured that the key informants selected for the study possessed the requisite knowledge and expertise to provide meaningful insights into the subject matter. This rigorous selection process was integral in securing informed and engaged participants who could contribute valuable perspectives to the study.

The participants were drawn from the following governmental, non-governmental and civil service organizations providing child and family health and welfare services or involved in decision-making on services for families in Botswana: Ministry of Local Government and Rural Development (Family Welfare Services Division under the Department of Social Protection), the United Nations International Children's Emergency Fund (UNICEF), the United Nations Educational, Scientific, and Cultural Organization (UNESCO), Raising Education Within Africa (REWA), Stepping Stones International (SSI), Global Communities, Childline Botswana, Sensobaby, Botswana Christian Health and AIDS Intervention Program (BOCAIP), Parent Effectiveness Training, Men and Boys for Gender Equality (MBGE), and SOS Children's Village Botswana and Marang Child Care Network.

By involving key stakeholders from governmental, non-governmental, and civil service organizations, the study was able to capture a diverse range of perspectives on child and family health and welfare services in Botswana. Before participating in the study, key informants were required to complete a consent form, indicating their willingness to be involved. A study information sheet was also provided to give participants a clear understanding of the purpose of the study and what would be expected of them during the interviews. This transparent approach to recruitment and informed consent ensured that participants were able to make an informed decision about their involvement in the study. The interviews with key informants were conducted between September 2021 and January 2022 and were audio recorded for accuracy. These recordings were later transcribed and securely stored in a shared One drive to protect the confidentiality and integrity of the data collected. Personal information was anonymized and kept in password-protected files, with access restricted to authorized personnel only. Processed data was stored on secure online servers hosted by the University of Cape Town, further ensuring its protection.

The Department of Social Protection (Family Welfare Services Division) in the Ministry of Local Government and Rural Development led this study in collaboration with Stepping Stones International, a non-governmental organization working in adolescent social protection on the Parenting for Lifelong Health Scale-Up of Parenting Evaluation Research (PLH-SUPER) (11). The Parenting for Lifelong Health program is a suite of evidence-informed parenting interventions designed to reduce child and adolescent maltreatment in resource-constrained countries (11).

## Ethics

Ethical approvals were obtained from the Universities of Cape Town (PSY2017-040) and Oxford (SPICUREC1a 20_015) and the Botswana Health Research and Development Committee. All participants were informed of the study objectives and subsequently consented to participate.

## Data preparation and analysis

Data were transcribed verbatim by TM and JK and analyzed by HG, TM, and JK. TM and JK were extensively trained in qualitative data analysis methods before they began the coding process. The development and application of the framework in our study were informed by several key elements. To begin with, the choice of the framework analysis approach was based on its relevance in policy research and its accessibility to multidisciplinary teams (14). This ensured that the framework would be appropriate for our research context and easily understood by all team members involved in the study. Additionally, the decision to use the EPIS framework to guide our interview guide further solidified the framework's applicability to our research questions. One of the main strengths of the framework analysis approach is its ability to accommodate both inductive and deductive reasoning (14). By utilizing this approach, we were able to ensure that our analysis was grounded in theoretical frameworks while also being responsive to the unique perspectives and experiences of our participants. The framework development stage was crucial in organizing the coded data into relevant categories and themes, guiding data interpretation and analysis. Regular meetings and discussions were held among the team members to ensure consensus in coding decisions, with the assistance of a third party whenever necessary. The framework not only informed the development and application of the coding scheme but also facilitated data comparison and consistency in the analysis process.

**Figure 1:**
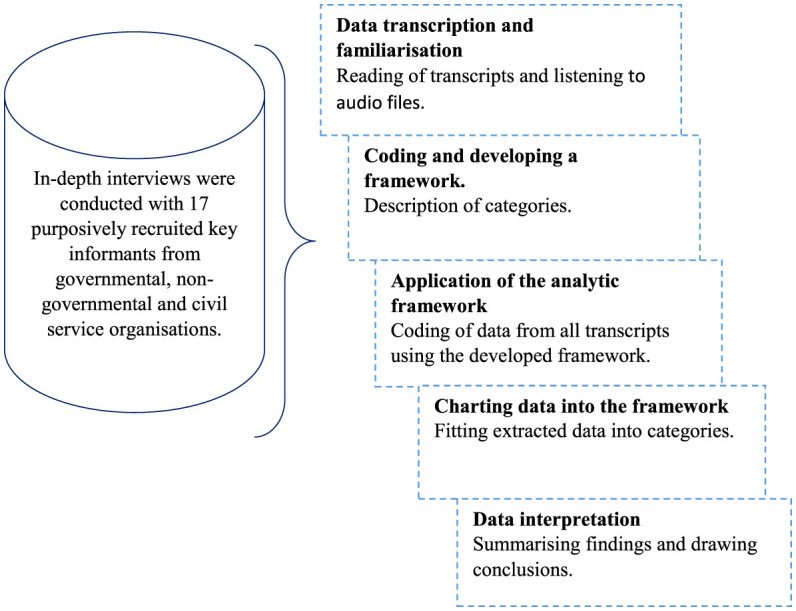
Study framework analysis.

The research complied with COREQ guidelines to enhance the validity and reliability of the study. This was achieved by ensuring transparency and thoroughness in reporting the research process. A completed form outlining the research team characteristics, steps followed in the study design, data analysis, and findings has been included as an Appendix to provide a clear overview of the study method (15). The framework analysis method (15) shown in Figure 1 was used for data management and analysis. It followed a seven-stage process; namely, (1) data transcription, (2) familiarization with interview transcripts, (3) coding, (4) developing a systematic and explicit framework to guide the analysis process, (5) application of the analytical framework to all transcripts, (6) charting data into the framework matrix, and (7) data interpretation (16). This method was chosen due to its flexibility and systematic nature making it accessible to researchers with diverse skills in qualitative research (17). A multidisciplinary team, including program implementers, research assistants and experienced researchers met bi-weekly throughout the data analysis process to discuss and judge interpretations derived from the data. The framework approach assisted in the development of a graphical hierarchical structure, with codes representing the most basic unit of analysis, followed by themes and subthemes that emerged from the data. By visualizing the connections between codes, themes, and subthemes, we were able to identify patterns and relationships within the data, leading to the generation of meaningful insights.

## Results

The participants shared their perspectives on antecedents and precedents to successfully implementing parenting interventions in Botswana. Although we report them using the EPIS model, broadly the results reveal the need for governmental involvement, harmonization of programs, instrumental participation of communities, networking, having competent human resources and adequate financial resources.

Table 2 shows study results on stakeholders' perspectives regarding the necessary conditions for successfully implementing parenting interventions in Botswana. The findings are presented in line with the EPIS framework components and phases. The study findings suggest that a comprehensive, collaborative, and multifaceted approach is necessary to promote sustainable positive parenting support. This approach should include (1) external factors, such as creating an enabling environment, aligning donor-program priorities and engaging stakeholders; (2) innovative factors, such as adapting programs, empowering local communities and using a mixed approach; (3) bridging factors, such as collaboration among various organizations; and (4) intra- organizational factors, such as ensuring adequate organizational resources, program sustainability and staff support. These factors should be integrated to find lasting and scalable solutions. Our findings recommend integrating parent support programs into government and public health initiatives to prevent violence against children and promote child wellbeing.

**Table 2:** Key findings on stakeholders' perspectives regarding the necessary conditions for successfully implementing parenting interventions in Botswana.

**EPIS phases**	**EPIS components**	**Main themes**	**Sub-themes**
•Exploration •Preparation •Implementation •Sustainment	External context	Government support	- Formulation of family policies and guidelines - Government involvement in program delivery
		Donor priorities vs. program needs	- Harmonization of donor-programme priorities - Alignment of donor-programme needs/expectations
		Stakeholder engagement	- Local stakeholder involvement (including caregivers and children) - Building trusting relations with local organizations
•Preparation •Implementation	Innovative factors	Program adaptation	- Context-specific programs - Programme sustainability
		Local empowerment	- Capacity building - Programme ownership
		Mixed approaches	- Population-wide interventions - Specific interventions
•Exploration •Preparation •Implementation •Sustainment	Bridging factors	Collaborative networks	- Networking with other organizations - Resource sharing
•Preparation •Implementation •Sustainment	Inner context	Organizational resources	- Human and financial resources - Mental health support for staff

## External factors government support

The participants discussed several enabling factors, including government support for parenting programs. The suggestion on how the government could support the programs included making policy interventions. In addition, participants were of the view that the chances of program sustainability increase when the government takes a leading role in the implementation of parenting programs as opposed to depending on donor support:

“*Most of the programs that we do are donor funded and not sustainable, it goes back to my point of really consulting with the government so that the government can actually adopt and implement this” (Imp_01)*.

The participants raised the need for government to set up policy translation and implementation frameworks to augment the work that is already being done by organizations implementing parenting programs. Others mentioned the need for family policies and guidelines:

“*I think this is where we are lacking as a country, we really need to put in place all these processes that allow us to have guidelines and models, that all of us have sat down and agreed on as stakeholders who are working with children”—(Nongov_03)*.

### Donor relations

Some of the participants spoke about how the conditions set in donor requirements may at times impede the successful implementation of parenting programs. Several participants expressed concern regarding the mismatch in donor and program expectations:

“*For programs tasked to deliver objectives and goals set by funders, implementers have no choice but to make promises to communities which are beyond the sustainable scope”—(Imp_03)*.

The participants encouraged collaboration between donors and program implementers to align priorities and promote the implementation of exceptional and effective parent support programs.

### Stakeholder engagement

One of the most critical processes in program implementation is stakeholder identification and management. As such the need for stakeholder consultations to build trusting relations with local authorities and gather useful information for implementation was stressed by most participants:

“*In Botswana, we have areas where there are rampant issues of child abuse and child marriages. If you go to those areas as an outsider trying to address those issues along with human rights perspectives and children's rights issues, the community will question your approach.”—(Gov_01)*.

The results also reveal that stakeholder engagement is considered very important in Botswana. A participant described Botswana as a consultative nation requiring a bottom- up consultative process.

“*Uhm, I think as a country they are very consultative nation. Uhm, nothing ever goes in this country without consultations …. first and foremost, community leaders. It needs to be sort of a bottom-up approach. I know that in most cases …we tend to want to turn things around and do an up-bottom approach, which most of the time does not work.”—(Imp_06)*.

The urge from participants was that parenting programs should be combined with existing social systems and structures that honor local leadership and exalt the instrumental participation of ordinary citizens.

## Innovation factors

The results show that implementers of parenting programs in Botswana believe that innovative approaches are key to program success. The participants considered agility, community ownership and instrumental participation to be key.

### Program sustainability

Interviewees opined that process monitoring and evaluation were essential for sustainability. And that sustainability of the program is not serendipitous but should be planned for with the context in mind:

“*A lesson learnt is about having a sustainability plan. Most of the time people come up with interventions and zero sustainability plans: You know, we just hope that as things unfold, the program will find feet and then it will continue, you know, and we don't actually sit down and say, how do we envision this working out in the event that we pull out of the whole process?”– (Nongov_01)*.

The participants also mentioned the need to adapt programs to suit post-COVID changes:

“*COVID-19 has changed the contextual framework to deliver parenting programs. Programs should be aware of contextual changes e.g., communication, farming, transportation, and technological Innovations are different across provinces in Botswana and these differences impact parenting availability of community members to engage in parenting programs.”—(Gov_02)*.

The participants viewed the imposition of programs and lack of consultation as a hindrance to program sustainability.

“*What has not worked I think it's trying to impose some programs on people, especially communities, because they will reject them outright.”—(Imp_04)*.

A participant from the non-governmental sector bemoaned poor community consultation and involvement. Attributing program failure and poor utilization and wastage of resources to it:

“*I think Botswana is probably one of the best countries to do a case study on the epic amount of failures of programs, the short-term success of programs, I think we could probably win an award. We have had so many from the government level to the private sector to NGO, we have had so many incredible programs, curricula, training, capacity building… and when I say millions, I mean millions of U.S. dollars spent. And there is no evidence for it anywhere. I really hate to say it, but no evidence for it anywhere, and I think that the biggest reason for this is that somebody is sitting in an office who has a great idea and has decided to go ahead and implement it without engaging the community in advance, and without giving ownership to the community to sustain it.”—(Nongov_2)*.

Similarly, some participants associated the rigidity of parenting interventions with their failure:

“*When we reviewed the program, we wanted to make a lot of adjustments because it was developed outside, however, we were going to compromise the quality of the program. So, we decided the program would not work here in Botswana and it did not survive”—(Imp_05)*.

Programs delivered through many sessions were perceived as difficult to implement and said to experience challenges such as poor attendance and low participant retention.

### Community ownership

Contrary to imposition, community ownership of programs was seen as central to the continuity of parenting interventions:

“*This is a difficult one, for us to be able to provide sustainable services, we need to ensure that there is ownership, and here when you talk about ownership, you must start with influential people like the community leaders. If they don't like something that you come up with, it will never see the day. Therefore, they are the people who will help you to talk to their communities about the services that you want to provide. If we leave them out, don't think anything will work.”—(Nongov_04)*.

Program ownership was described as a process that requires a detailed understanding of beneficiaries' experiences, needs and suggestions to create a program that adds value to the community. Others felt local empowerment could only be achieved when communities are placed at the center of the program agenda:

“*They need to see you as somebody who is coming to put through your agenda into their lives. Because if they perceive it as your agenda, they will expect you to reward them for pushing your agenda.”—(Gov_3)*.

The participants encouraged the prioritization of input from policymakers and stakeholders including the beneficiaries of the program:

“*Input from policy makers, stakeholders and gatekeepers should be prioritized by program developers and implementers. Stakeholder meetings must also include the beneficiaries of programs (such as adolescents and male caregivers).”—(Nongov_05)*.

### Program reach

The use of population-wide interventions (e.g., radio programs) and interventions targeting individuals at risk and those living in remote areas were recommended by the participants.

“*Parent support programs should aim to reach all parents including those living in remote areas and those who have diverse needs such as single parents and caregivers of children with special needs.”—(Gov_04)*.

However, two participants from non-governmental organizations highlighted challenges related to digital parenting interventions, citing connectivity issues and the lack of mobile devices as barriers to providing remote parenting interventions in rural Botswana.

## Bridging factors

The participants opined that a siloed approach to implementing parenting programs leads to donor/researcher fatigue, poor utilization of resources and subsequent failure. Therefore, the formation of collaborative networks among implementing partners, policymakers and stakeholders was recommended to efficiently deliver efficient parenting services:

“*…the organizations need to work together and share lessons and resources such as assessment tools… platforms must be provided for implementers and stakeholders to meet regularly.”—(Imp_08)*.

The lack of collaboration among stakeholders was noted to be overwhelming to communities:

“*I think you know we are confusing communities. We were overwhelming them. So, I think we need to come together and then leverage on each other's advantages, and make sure that you know, even the communities are aware of our activities, and how best they can support to deliver the programs to the communities.”—(Nongov_03)*.

## Intra-organizational factors

Several participants highlighted the need to implement sustainable parent support programs. A few participants suggested the integration of parenting and social welfare services “ to overcome program sustainment challenges related to limited resources:

“*If we get the coordination right, then it means we can see how we mainstream all these elements into what government is providing for sustainable funding.”—(Imp_2)*.

Coupled with collaborative partnerships, participants also spoke of the need to prioritize the wellbeing of staff working for implementing organizations. They raised concerns about the risk of overburdening existing staff. According to participants, the organizations' support to staff members should include the provision of mental health services to boost staff motivation:

“*Facilitators must receive mental health support in addition to other incentives so that they remain motivated in doing their work.”—(Imp_07)*.

## Discussion

The study's findings align with previous research calling for governmental support for evidence-based parent support programs and the development of family and child welfare policies (18, 19). Additionally, research has emphasized the importance of collaboration among researchers, practitioners, and policymakers to enhance public health program dissemination and parenting interventions. Evidence from Botswana highlights the need for multi-stakeholder collaboration for the successful implementation and sustainability of parenting programs. This collaboration should be based on a “power with” rather than a “power over” relationship, meaning it should be participatory rather than coercive (20).

The study results indicate that consultation with and input from communities is vital for successful program redesign that meets their needs and expectations. Baumann et al. (21) have similarly emphasized the importance of stakeholder involvement in parenting intervention design and implementation for program success and sustainability. Thus, it is essential to create feedback loops that facilitate evidence-based program changes to guide ongoing improvement in program delivery and sustainability (22).

Experience from Botswana implementers also shows that parenting programs run the risk of becoming utopian concepts meant for researchers and tick-box exercises for donors. The urge is for stakeholders, including donors, the government, implementing partners and communities to find ways of working together on shared and overlapping objectives. Holistic stakeholder engagement is critical at all stages of parenting implementation. The findings back up previous research that found that the success of any project run in collaboration with communities is ultimately determined by how it is managed and the relationships that exist among and between the collaborating partners (21, 23).

During the study, the participants noted that though local leadership was highly respected in Botswana communities, decisions and programs should not be imposed upon them. Community leaders play a vital role in mobilizing communities in most indigenous areas and previous research has shown that involving local leadership, especially through community advisory boards can help develop relevant parenting interventions and encourage parental and guardian participation (24). Additionally, local leaders are often the gatekeepers of local cultures, which can facilitate the implementation of culturally responsive interventions.

Although a “power with” relationship is preferred, the study's results suggest that acknowledging the different levels of influence and power among stakeholders is necessary. The redistribution of power between researchers and communities should not be misconstrued as an opportunity to interfere with societal power relationships, particularly in communities where tribal hierarchies are observed (25). Instead, partners should prioritize humility to build unity and facilitate collaboration among stakeholders from scientific, economic, and cultural backgrounds (26). For instance, successful program implementation often depends on the involvement of local leaders or structures in negotiating community entry as well as governmental support in providing financial resources, adopting program operations, and establishing the necessary policies and legal framework.

The critical role of governments in bridging the gap between practice and research has been extensively documented (21, 27, 28). Previous studies have recommended that policymakers establish strong relationships with researchers and other stakeholders to effectively fulfill this role (29–31). Our study reinforces the need to engage with governments to implement effective parenting interventions. As superintendents of policy and legislation, governments play a crucial role in safeguarding the rights of children and adolescents as a vulnerable group in society. As a result, our results indicate that implementers are compelled to enhance their efforts on policy translation and implementation. McKay et al. (32) argue that implementation scientists should collaborate with policymakers, considering that policy recommendations need to be organized swiftly and delivered in accessible languages.

According to the study's participants, adequate human and financial resources are essential for successful program implementation. These findings align with previous research on implementation barriers, such as insufficient funding, inadequate planning and limited consideration of sustainability and scalability (14, 33). Our study also highlights the importance of mental health support for program facilitators and those providing family and child welfare services. Previous research has reported challenges that health and social welfare practitioners face globally, including high staff turnover rates, job dissatisfaction, stress, and burnout. These challenges have been exacerbated amid the COVID-19 pandemic, particularly in LMICS, where practitioners are experiencing increased workloads, longer work hours, and limited resources (34, 35).

## Limitations, implications, and future research

Methodological limitations to our study include the potential for bias in the selection of participants. Since our study focused on stakeholders' perspectives, it may lack the input of the actual parents who would be the recipients of these interventions. This could impact the generalizability of our findings. In terms of future directions, further research should seek to include the voices of parents and families themselves to gain a more comprehensive understanding of the necessary conditions for successful parenting interventions in Botswana.

Our study highlights the importance of comprehensive stakeholder engagement, including government officials and representatives from non-governmental organizations, in the planning and implementation of parent support programs. This collaborative approach ensures that programs are relevant, sustainable, and effectively meet the needs of the community. Furthermore, our study emphasizes the importance of adapting parenting interventions to the local context, taking into consideration the cultural norms and resources available in Botswana. This suggests that policymakers should invest in culturally responsive programs that are agile and flexible to meet the diverse needs of families in the country. Public-private partnerships are also identified as a key factor in ensuring the sustainability of parenting interventions in Botswana. Policymakers should prioritize building and maintaining these partnerships to leverage resources and expertise from both sectors.

Future research in this area could explore the impact of involving families and communities in the design and delivery of parenting interventions. Longitudinal studies could track the progress of families over time to determine the long-term effects of these interventions on parenting practices and child outcomes. Additionally, research could focus on the role of social support networks in promoting positive parenting behaviors and strengthening family relationships. By involving a diverse range of stakeholders, including parents, policymakers, and community members, future research can provide a more comprehensive understanding of the factors that contribute to effective parenting interventions in Botswana.

## Conclusion

Our study included key stakeholders, comprising officials from government and non- governmental organizations responsible for overseeing and implementing parent support programs and policies in Botswana. By involving these critical stakeholders, we gained rich insights and diverse experiences that informed our findings. Our study emphasized the importance of program implementation adaptations necessary for achieving success and sustainability in a low-resource setting. It highlighted the critical need for engaging stakeholders comprehensively and consistently to maintain program ownership, reach and relevance. Public-private partnerships are key to ensuring program sustainability, although organizational factors are critical in facilitating effective program delivery. Our findings underscore the need for agile and context specific parenting interventions. That remain connected to the communities they serve. The study suggests that communities and primary beneficiaries of parenting interventions should be active participants in all phases, including design, implementation, monitoring, and evaluation. Ideally, parent support programs should be learning programs that draw upon emerging evidence to improve parenting outcomes.

## Author's Note - Positionality Statement

Following the pre-specified research questions guided the exploration of the implementation experiences of participants in the current study. The research questions focused on the implementation of services rather than the personal and family life experiences of participants. To support the integrity of the research, the participants were informed at the beginning of every interview that the research team was interested in understanding implementation experiences, not in evaluating the work of the organizations, and that all opinions were welcome. The research team were independent of the Botswana NGO and Government employees. Researchers in this study have collaborated with program implementers in diverse settings and have been studying parenting programmes for several years. Frequent discussions and reflection were used to support the integrity of the study.

## Data availability statement

The raw data supporting the conclusions of this article will be made available by the authors, without undue reservation.

## Ethics statement

The studies involving humans were approved by Universities of Cape Town (PSY2017-040), Oxford (SPICUREC1a__20_015) and the Botswana Health Research and Development Committee. The studies were conducted in accordance with the local legislation and institutional requirements. The participants provided their written informed consent to participate in this study.

## Author contributions

HG: Conceptualization, Data curation, Formal analysis, Investigation, Methodology, Writing – original draft. TM: Data curation, Formal analysis, Validation, Writing – review & editing. JK: Data curation, Formal analysis, Investigation, Writing – original draft. JR: Data curation, Methodology, Project administration, Resources, Writing – review & editing. SJ: Methodology, Supervision, Validation, Writing – review & editing. LJ: Investigation, Project administration, Validation, Writing – review & editing. NM: Data curation, Investigation, Validation, Writing – review & editing. MM: Funding acquisition, Resources, Validation, Writing – review & editing. MMN: Funding acquisition, Investigation, Resources, Validation, Writing – review & editing. BN: Writing – review & editing. YS: Formal analysis, Methodology, Writing – review & editing. JL: Conceptualization, Funding acquisition, Resources, Supervision, Writing – review & editing. LC: Funding acquisition, Resources, Supervision, Writing – review & editing. CW: Conceptualization, Funding acquisition, Resources, Supervision, Writing – review & editing.
